# Development and External Validation of a Model Predicting New‐Onset Chronic Uveitis at Different Disease Durations in Juvenile Idiopathic Arthritis

**DOI:** 10.1002/art.42329

**Published:** 2022-12-13

**Authors:** Joeri W. van Straalen, Lianne Kearsley‐Fleet, Jens Klotsche, Sytze de Roock, Kirsten Minden, Arnd Heiligenhaus, Kimme L. Hyrich, Joke H. de Boer, Lovro Lamot, Alma N. Olivieri, Romina Gallizzi, Elzbieta Smolewska, Enrique Faugier, Serena Pastore, Philip J. Hashkes, Cristina N. Herrera, Wolfgang Emminger, Rita Consolini, Nico M. Wulffraat, Nicolino Ruperto, Joost F. Swart, the Paediatric Rheumatology International Trials Organisation (PRINTO), UK CAPS study, and German ICON study, G Cleary, E Baildam, L Wedderburn, J Davidson, A Chieng, F McErlane, H Foster, C Ciurtin, Y Ioannou, W Thomson, K Hyrich, Tilmann Kallinich, Frank Dressler, Jasmin Kümmerle Deschner, Frank Weller‐Heinemann, Gerd Horneff, Anton Hospach, Kirsten Mönkemöller, Johannes Peter Haas, Daniel Windschall, Ivan Foeldvari, Dirk Foell

**Affiliations:** ^1^ Department of Pediatric Immunology and Rheumatology, Wilhelmina Children's Hospital, University Medical Center Utrecht, and Faculty of Medicine Utrecht University Utrecht The Netherlands; ^2^ Centre for Epidemiology Versus Arthritis The University of Manchester Manchester UK; ^3^ Epidemiology Unit German Rheumatism Research Centre Berlin Berlin Germany; ^4^ Epidemiology Unit, German Rheumatism Research Centre Berlin, and Department of Pediatric Respiratory Medicine, Immunology and Critical Care Medicine, Charité Universitätsmedizin Berlin, corporate member of Freie Universität Berlin and Humboldt‐Universität zu Berlin Berlin Germany; ^5^ Department of Ophthalmology, St. Franziskus Hospital, Münster, Germany, and University of Duisburg‐Essen Essen Germany; ^6^ Centre for Epidemiology Versus Arthritis, The University of Manchester, and NIHR Manchester BRC, Manchester University NHS Foundation Trust Manchester Academic Health Science Centre Manchester UK; ^7^ Department of Ophthalmology University Medical Center Utrecht Utrecht The Netherlands; ^8^ Sestre Milosrdnice University Hospital Center Zagreb, Zagreb, Croatia, and University of Zagreb School of Medicine Zagreb Croatia; ^9^ Dipartimento della Donna del Bambino e di Chirurgia Generale e Specialistica Università degli Studi della Campania L.Vanvitelli Naples Italy; ^10^ Department of Medical of Health Sciences Magna Graecia University Catanzaro Italy; ^11^ Department of Pediatric Cardiology and Rheumatology Medical University of Lodz Lodz Poland; ^12^ Dipartimento della Donna del Bambino e di Chirurgia Generale e Specialistica Università degli Studi della Campania L.Vanvitelli Naples Italy; ^13^ Institute for Maternal and Child Health, IRCCS Burlo Garofolo Trieste Italy; ^14^ Pediatric Rheumatology Unit Shaare Zedek Medical Center, and Hebrew University Hadassah School of Medicine Jerusalem Israel; ^15^ Servicio de Reumatología, Hospital de Niños Roberto Gilbert Elizalde Guayaquil Ecuador; ^16^ Department of Pediatrics University Children's Hospital, Medical University of Vienna Vienna Austria; ^17^ Division of Pediatrics, Department of Clinical and Experimental Medicine University of Pisa Pisa Italy; ^18^ Clinica Pediatrica e Reumatologia, IRCCS Istituto Giannina Gaslini Genoa Italy

## Abstract

**Objective:**

To develop and externally validate a prediction model for new‐onset chronic uveitis in children with juvenile idiopathic arthritis (JIA) for clinical application.

**Methods:**

Data from the international Pharmachild registry were used to develop a multivariable Cox proportional hazards model. Predictors were selected by backward selection, and missing values were handled by multiple imputation. The model was subsequently validated and recalibrated in 2 inception cohorts: the UK Childhood Arthritis Prospective Study (CAPS) study and the German Inception Cohort of Newly diagnosed patients with juvenile idiopathic arthritis (ICON) study. Model performance was evaluated by calibration plots and C statistics for the 2‐, 4‐, and 7‐year risk of uveitis. A diagram and digital risk calculator were created for use in clinical practice.

**Results:**

A total of 5,393 patients were included for model development, and predictor variables were age at JIA onset (hazard ratio [HR] 0.83 [95% confidence interval (95% CI) 0.77–0.89]), ANA positivity (HR 1.59 [95% CI 1.06–2.38]), and International League of Associations for Rheumatology category of JIA (HR for oligoarthritis, psoriatic arthritis, and undifferentiated arthritis versus rheumatoid factor–negative polyarthritis 1.40 [95% CI 0.91–2.16]). Performance of the recalibrated prediction model in the validation cohorts was acceptable; calibration plots indicated good calibration and C statistics for the 7‐year risk of uveitis (0.75 [95% CI 0.72–0.79] for the ICON cohort and 0.70 [95% CI 0.64–0.76] for the CAPS cohort).

**Conclusion:**

We present for the first time a validated prognostic tool for easily predicting chronic uveitis risk for individual JIA patients using common clinical parameters. This model could be used by clinicians to inform patients/parents and provide guidance in choice of uveitis screening frequency and arthritis drug therapy.

## INTRODUCTION

Juvenile idiopathic arthritis (JIA) is defined as arthritis of unknown cause lasting for >6 weeks in a child younger than 16 years (1). JIA is the most common form of chronic rheumatic illness in childhood worldwide with an incidence estimated to be 1.6–23 cases per 100,000 children (2). On average, 13% of JIA patients develop uveitis (3), an intraocular inflammation which can lead to serious complications including loss of vision if not treated in a timely manner (4, 5). Chronic uveitis with insidious onset of flares is the most common form of JIA‐related uveitis and usually does not present with apparent symptoms until ocular complications arise (6, 7). For this reason, JIA patients should be screened by an ophthalmologist, and several guidelines for the frequency and duration of this screening exist (7, 8, 9, 10, 11).

Current screening guidelines differentiate patients according to a roughly high, moderate, or low risk of developing uveitis, which are subjective terms that could be interpreted differently by individual clinicians. To date, pediatric rheumatologists do not have a comprehensive and validated tool for obtaining absolute risk estimates for chronic uveitis based on characteristics of individual JIA patients.

The objectives of this study were to 1) develop a prediction model for new‐onset chronic uveitis in JIA that could be of assistance in clinical practice, and 2) validate this model in 2 external cohorts.

## PATIENTS AND METHODS

### Patients

Data from the international Pharmachild registry were used for developing the prediction model. Pharmachild is an ongoing pharmacovigilance project that started in 2011 with the objective of monitoring adverse events in JIA patients receiving drug therapy (12). Inclusion criteria are children with JIA according to International League of Associations for Rheumatology (ILAR) criteria (13) who are receiving treatment or were previously treated with nonsteroidal antiinflammatory drugs (NSAIDs), glucocorticoids, or conventional synthetic or biologic disease‐modifying antirheumatic drugs (DMARDs). Patients are included from 85 Paediatric Rheumatology International Trials Organisation (PRINTO) centers from 31 countries (14).

Data were obtained up to May 3, 2019. Only patients with ≥2 registered visits were included in the current study. Exclusion criteria were enthesitis‐related arthritis (ERA), systemic arthritis, rheumatoid factor (RF)–positive polyarthritis, uveitis prior to JIA onset, a diagnosis of acute uveitis, and an unknown date of uveitis diagnosis. ERA patients were excluded because of probable acute uveitis onset (4). Systemic and RF+ polyarthritis patients were excluded because these conditions are known to have low risk for uveitis development (9). RF+ patients from other ILAR categories were not excluded.

### Outcomes and predictors

The outcomes predicted in this study were the 2‐, 4‐, and 7‐year risk of new‐onset chronic uveitis after onset of JIA. These time points are thresholds for disease duration in current screening guidelines (9). For all patients, a first diagnosis of chronic uveitis was determined from free‐text fields and tick boxes filled in at registration into Pharmachild or adverse events reported using the Medical Dictionary for Regulatory Activities coding system (version 22) during follow‐up. Dates of therapy change due to uveitis were not used as uveitis diagnosis dates. All uveitis event descriptions were reviewed by 3 researchers (JvS, SdR, JS) to ensure acute and posterior cases were excluded.

Potential predictors of uveitis were identified by consensus of the researchers and the existing literature. For each patient, if available, the following information was collected: sex, age at JIA onset, ILAR category of JIA, antinuclear antibody (ANA) status, HLA–B27 status, RF status, family history of autoimmune disease in first‐ and second‐degree relatives (yes/no), and geographic region. Patients were grouped into the following geographic regions based on the country of the center in which they were treated: Western Europe, Central and Eastern Europe, Scandinavia, Southern Europe, and other region (15). The latter category included patients from Latin America, Africa, and Asia, and had to be analyzed as a whole due to few events of uveitis. An overview of included countries and corresponding regions is provided in Supplementary Table 1, available on the *Arthritis & Rheumatology* website at https://onlinelibrary.wiley.com/doi/art.42329. Onset date of JIA was defined on the Pharmachild case report forms as the “date of occurrence of the first clinical manifestation consistent with the disease.” Age at JIA onset was treated as a continuous variable.

Methotrexate (MTX) and adalimumab (ADA) therapy are effective in the treatment of uveitis in JIA (4, 8). Therefore, we also collected data on MTX and ADA use and discontinuation prior to uveitis onset to study a possible protective effect. These variables were not considered for inclusion into the prediction model since it is not possible to determine whether a newly diagnosed JIA patient will receive ADA or MTX and we wanted our prediction model to make uveitis predictions early in the disease course.

### Model development

Variables collected were first analyzed in a univariable Cox proportional hazards regression analysis. Variables were considered statistically significant if the 95% confidence interval (95% CI) of the hazard ratio (HR) did not contain 1. Missing values were handled by multiple imputation using chained equations (16). Estimates for 20 imputed data sets were pooled using Rubin's rules. Subsequently, all variables were entered into a multivariable Cox prediction model and removed by stepwise backward selection in the multiple imputed data sets with a *P* value threshold of 0.15. To avoid overfitting and poor performance of the prediction model during the external validation, we decided a priori to create risk groups of ILAR categories with similar risks of developing uveitis. Based on 2 large‐scale studies, we grouped together RF– polyarthritis versus psoriatic, undifferentiated, and oligoarticular arthritis (9, 17). The proportional hazards assumption was checked in the twentieth imputed data set by testing for independence of the Schoenfeld residuals over time, and linearity of continuous variables was checked by plotting these against the Martingale residuals.

### External validation

For external validation and subsequent model recalibration, data from 2 JIA inception cohorts were used, and the same exclusion criteria were applied.

The Childhood Arthritis Prospective Study (CAPS), established in 2001, is a UK prospective inception cohort study of children with new‐onset idiopathic inflammatory arthritis in childhood (18). Children are recruited within 6 months of first presentation to pediatric rheumatology from 1 of 7 tertiary care UK rheumatology centers if they are <16 years of age with new‐onset arthritis in ≥1 joint lasting for ≥2 weeks. Baseline data are collected from clinical records and include demographic information, disease duration, ILAR category, clinical markers of disease, current medication, JIA core outcome variables, and information on uveitis diagnosis and treatment. Patients are followed up annually for 5 years, with additional data collected at 7 and 10 years. Follow‐up information includes disease activity, ILAR category, changes in medication, and information on uveitis.

The Inception Cohort of Newly diagnosed patients with juvenile idiopathic arthritis (ICON) is a multicenter, controlled cohort study (19). Patients were enrolled within 12 months after a diagnosis of JIA according to ILAR criteria at 11 of the largest pediatric rheumatology centers in Germany from 2010 to 2014 and have been followed up since then. At first presentation and inclusion in ICON, demographic information, disease duration, ILAR category, clinical markers of disease, current medication, history of uveitis, and JIA core outcome variables are reported. Follow‐up information on clinical markers of disease, current medication, diagnosis of uveitis, and JIA core outcome variables were collected every 3 months during the first year and then every 6 months thereafter.

### Model validation and recalibration

For external validation, coefficients of the prediction model and the mean linear predictor in the imputed Pharmachild data sets were transferred to the analysts for CAPS (LKF) and ICON (JK), and linear predictors were calculated for all patients in the validation data sets (20). The prediction model was recalibrated in 2 ways: 1) by determining the 2‐, 4‐, and 7‐year baseline survival probabilities in the validation cohorts after fitting a Cox regression with the linear predictors as the only parameter (i.e., “recalibration in the large”), and 2) by using the coefficient of this model as a shrinkage factor for the linear predictors (i.e., “logistic recalibration”) (21, 22). Performance of the recalibrated prediction models in the validation cohorts was assessed for the 2‐, 4‐, and 7‐year risk of chronic uveitis by means of the corresponding C statistic and calibration plots. The C statistic ranges from 0.5 to 1 and indicates how well a model can distinguish patients who will develop the predicted outcome from patients who will not (23). For the calibration plots, observed probabilities or Kaplan‐Meier estimates of chronic uveitis within quintiles of the validation data were plotted against the mean predicted probabilities. The recalibrated model that demonstrated best calibration in both validation cohorts was presented as our final prediction model.

To compare discriminative ability of our model to current uveitis screening guidelines, we also determined the C statistics for a model based on parameters from the modifications of the American Section of Rheumatology and Ophthalmology screening guidelines made by Heiligenhaus et al (9). All analyses were performed with R version 4.0.0 (24) and the stats, rms, survival, psfmi, and Hmisc packages. We adhered to the Transparent Reporting of a multivariable prediction model for Individual Prognosis Or Diagnosis (TRIPOD) guidelines (25).

## RESULTS

### Cohort characteristics

Initially, 2,756 patients were excluded (2,244 because of ERA, systemic arthritis, or RF+ polyarthritis diagnosis), leaving 6,186 patients (Supplementary Figure 1, https://onlinelibrary.wiley.com/doi/art.42329). New‐onset uveitis had occurred in 900 patients (14.5%); however, another 793 of these patients were also excluded from further analysis because of an unknown date of uveitis diagnosis (see Supplementary Table 2 for the characteristics of all 900 uveitis patients at https://onlinelibrary.wiley.com/doi/art.42329). A total of 5,393 Pharmachild patients were included for analysis, and 107 of these patients were diagnosed as having chronic uveitis, with a median time from JIA onset to uveitis diagnosis of 2.3 years (interquartile range [IQR] 0.7–4.5). Of the total 5,393 included patients, 74.3% were girls, and 36.0% were treated in Southern Europe. The most common ILAR categories were oligoarthritis (47.7%) and RF– polyarthritis (38.4%) (Table 1). Patients who developed chronic uveitis were younger at JIA onset than those who did not develop uveitis (median age 2.2 versus 5.0 years), were more often ANA positive (63.6% versus 44.5%), and were less likely to have RF– polyarthritis (27.1% versus 38.6%). Furthermore, patients who did not develop chronic uveitis had more often been treated with MTX (84.2% versus 65.4%) or ADA (15.3% versus 3.7%).

**Table 1:** Patient characteristics of the Pharmachild cohort used to develop a model for predicting new‐onset chronic uveitis in children with JIA*

Characteristic	Total cohort (n = 5,393)	No chronic uveitis (n = 5,286)	Chronic uveitis (n = 107)†	HR (95% CI)
Geographic region				
Southern Europe	1,943 (36.0)	1,912 (36.2)	31 (29.0)	Referent
Scandinavia	540 (10.0)	535 (10.1)	5 (4.7)	0.52 (0.20–1.34)
Western Europe	961 (17.8)	902 (17.1)	59 (5.5)	3.74 (2.40–5.81)‡
Central and Eastern Europe	1,432 (26.6)	1,422 (26.9)	10 (9.3)	0.47 (0.23–0.98)‡
Other	517 (9.6)	515 (9.7)	2 (1.9)	0.24 (0.06–1.03)
Girls	4,007 (74.3)	3,925 (74.3)	82 (76.6)	1.05 (0.67–1.65)
Age at JIA onset, median (IQR) years	4.9 (2.3–9.2)	5.0 (2.3–9.3)	2.2 (1.6–4.1)	0.81 (0.75–0.88)‡
ILAR category				
Oligoarthritis	2,575 (47.7)	2,517 (47.6)	58 (54.2)	Referent
Persistent	1,707 (66.2)	1,668 (66.3)	37 (63.8)	
Extended	870 (33.8)	849 (33.7)	21 (36.2)	
RF– polyarthritis	2,072 (38.4)	2,043 (38.6)	29 (27.1)	0.60 (0.38–0.95)‡
Psoriatic arthritis	259 (4.8)	251 (4.7)	8 (7.5)	1.30 (0.61–2.74)
Undifferentiated arthritis	487 (9.0)	475 (9.0)	12 (11.2)	1.10 (0.59–2.06)
Laboratory characteristics				
ANA positive§	2,309 (44.9)	2,241 (44.5)	68 (63.6)	2.09 (1.40–3.12)‡
RF positive¶	26 (0.5)	26 (0.5)	0 (0.0)	–
HLA–B27 positive#	348 (11.0)	339 (11.0)	9 (14.8)	1.24 (0.58–2.65)
Family history of autoimmune disease**	1,468 (28.2)	1,434 (28.2)	34 (31.8)	1.24 (0.82–1.88)
Family history of uveitis**	9 (0.2)	9 (0.2)	0 (0)	–
Drug therapy				
MTX prior to uveitis or last follow‐up	4,521 (83.8)	4,451 (84.2)	70 (65.4)	0.28 (0.19–0.42)‡
Duration from last MTX stop to uveitis diagnosis, median (IQR) years	–	–	0.9 (0.4–2.2)††	–
ADA prior to uveitis or last follow‐up	811 (15.0)	807 (15.3)	4 (3.7)	0.18 (0.06–0.49)‡
Duration from last ADA stop to uveitis diagnosis, median (IQR) years	–	–	2.7 (1.6–3.8)‡‡	–

* Except where otherwise indicated, values are number (%) of patients. Missing values were imputed via multiple imputation. JIA = juvenile idiopathic arthritis; HR = hazard ratio; 95% CI = 95% confidence interval; IQR = interquartile range; ILAR = International League of Associations for Rheumatology.

† Chronic uveitis patients only include cases with an available diagnosis date.

‡ Indicates statistically significant.

§ For the antinuclear antibody (ANA)–positive characteristic, total n = 5,141 (5,034 patients without chronic uveitis, 107 patients with chronic uveitis).

¶ For the rheumatoid factor (RF)–positive characteristic, total n = 4,821 (4,730 patients without chronic uveitis, 91 patients with chronic uveitis).

# For the HLA–B27 positive characteristic, total n = 3,153 (3,092 patients without chronic uveitis, 61 patients with chronic uveitis).

** Family history of autoimmune disease or uveitis characteristics include first‐ and second‐degree relatives. Total n = 5,198 (5,091 patients without chronic uveitis, 107 patients with chronic uveitis).

†† Duration of last methotrexate (MTX) stop to uveitis was calculated from 34 chronic uveitis patients.

‡‡ Duration of last adalimumab (ADA) stop to uveitis was calculated from 2 chronic uveitis patients.

Characteristics of patients in the CAPS and ICON cohorts with complete information for the prediction model variables are presented in Table 2. In the CAPS cohort, 88 (12.6%) of 700 included patients developed chronic uveitis. In the ICON cohort, 119 (15.7%) of 758 included patients developed chronic uveitis. For both cohorts, the median time from JIA onset to uveitis diagnosis was shorter than what was observed in the Pharmachild cohort (median years 2.1 [IQR 1.1–4.8] for CAPS and 1.0 [IQR 0.3–2.6] for ICON). Patients in the CAPS and ICON cohorts who developed chronic uveitis were more often ANA positive (81.8% for CAPS and 87.4% for ICON) and more often had oligoarthritis (61.4% for CAPS and 68.1% for ICON) compared to uveitis patients in the Pharmachild cohort (63.6% ANA positive and 54.2% with oligoarthritis).

**Table 2:** Patient characteristics of the CAPS and ICON cohorts used for external validation of the prediction model*

	CAPS cohort			ICON cohort		
Characteristic	Total cohort (n = 700)	No chronic uveitis (n = 612)	Chronic uveitis (n = 88)	Total cohort (n = 758)	No chronic uveitis (n = 639)	Chronic uveitis (n = 119)
Girls	475 (67.9)	410 (67.0)	65 (73.9)	547 (72.2)	456 (71.4)	91 (76.5)
Age at JIA onset, median (IQR) years	6.2 (2.5–10.5)	6.8 (2.9–10.8)	2.4 (1.6–5.3)	5.4 (2.5–10.3)	6.5 (2.9–11.0)	2.5 (1.7–3.7)
ILAR category						
Oligoarthritis	426 (60.9)	372 (60.8)	54 (61.4)	412 (54.4)	331 (51.8)	81 (68.1)
Persistent	378 (54)	332 (54)	46 (52)	339 (44.7)	271 (42.4)	68 (57.1)
Extended	48 (7)	40 (7)	8 (9)	73 (9.7)	60 (9.4)	13 (10.9)
RF– polyarthritis	182 (26.0)	160 (26.1)	22 (25.0)	239 (31.5)	208 (32.5)	31 (26.1)
Psoriatic arthritis	55 (7.9)	46 (7.5)	9 (10.2)	45 (5.9)	43 (6.7)	2 (1.7)
Undifferentiated arthritis	37 (5.3)	34 (5.6)	3 (3.4)	62 (8.2)	57 (8.9)	5 (4.2)
Laboratory characteristics						
ANA positive	386 (55.1)	314 (51.3)	72 (81.8)	450 (59.4)	346 (54.2)	104 (87.4)
RF positive†	28 (5.0)	24 (4.9)	4 (5.6)	23 (3.0)	19 (2.9)	4 (3.4)
HLA–B27 positive‡	32 (15.8)	28 (15.5)	4 (18.2)	70 (9.2)	68 (10.6)	5 (4.2)
Family history of autoimmune disease§	371 (53.0)	320 (52.3)	51 (58.0)	–	–	–
Family history of uveitis§	3 (0.4)	3 (0.5)	0 (0.0)	–	–	–
Drug therapy						
MTX prior to uveitis or last follow‐up	373 (53.3)	323 (52.8)	50 (56.8)	509 (67.2)	451 (70.6)	57 (47.9)
Duration from last MTX stop to uveitis diagnosis, median (IQR) years¶	–	–	2.1 (1.1–5.0)	–	–	1.0 (1.0–1.0)
ADA prior to uveitis or last follow‐up	42 (6.0)	37 (6.0)	5 (5.7)	88 (11.6)	88 (13.8)	0 (0)
Duration from last ADA stop to uveitis diagnosis, median (IQR) years#	–	–	5.3 (5.3–5.3)	–	–	–

* Except where otherwise indicated, values are number (%) of patients. See Table 1 for other definitions.

† For the RF‐positive characteristic, total Childhood Arthritis Prospective Study (CAPS) n = 562 (491 patients without chronic uveitis, 71 patients with chronic uveitis), and total Inception Cohort of Newly diagnosed patients with JIA (ICON) n = 758 (639 patients without chronic uveitis, 119 patients with chronic uveitis).

‡ For the HLA–B27 positive characteristic, total CAPS n = 203 (181 patients without chronic uveitis, 22 patients with chronic uveitis), and total ICON n = 758 (639 patients without chronic uveitis, 119 patients with chronic uveitis).

§ Family history of autoimmune disease and uveitis includes first‐ and second‐degree relatives. ICON does not collect data on familial autoimmune diseases.

¶ Duration of last MTX stop to uveitis was calculated from 9 chronic uveitis patients in the CAPS cohort and 1 chronic uveitis patient in the ICON cohort.

# Duration of last ADA stop to uveitis was calculated from 1 chronic uveitis patient in the CAPS cohort. No chronic uveitis patients in the ICON cohort had received ADA.

### Development of prediction model

On univariable analysis, ANA status (HR 2.09 [95% CI 1.40–3.12]) and age at JIA onset (HR 0.81 [95% CI 0.75–0.88]) were significantly associated with new‐onset chronic uveitis. RF– polyarthritis patients (HR 0.60 [95% CI 0.38–0.95]) had a significantly lower risk for developing uveitis compared to oligoarthritis patients, unlike psoriatic arthritis patients (HR 1.30 [95% CI 0.61–2.74]) and undifferentiated arthritis patients (HR 1.10 [95% CI 0.59–2.06]) who had higher risk of uveitis. Compared to patients from Southern Europe, Western European patients had a significantly higher risk of uveitis (HR 3.74 [95% CI 2.40–5.81]), and Central and Eastern European patients had a significantly lower risk of uveitis (HR 0.47 [95% CI 0.23–0.98]). Ultimately, the best combined predictors for new‐onset chronic uveitis development were age at JIA onset (HR 0.83 [95% CI 0.77–0.89]), ANA positivity (HR 1.59 [95% CI 1.06–2.38]), and ILAR category risk group (Table 3). Patients with oligoarthritis, psoriatic arthritis, or undifferentiated arthritis had a 1.40 times higher risk (95% CI 0.91–2.16) for developing uveitis over the study period compared to patients with RF– polyarthritis. The mean linear predictor in the Pharmachild data set for calculating a predicted probability of uveitis was –0.71.

**Table 3:** Strength of association of each variable used in the prediction model with new‐onset chronic uveitis in children with JIA*

Predictor variable	β	HR (95% CI)
Age at JIA onset	–0.19	0.83 (0.77–0.89)†
ANA positive	0.46	1.59 (1.06–2.38)†
ILAR category risk groups		
RF– polyarthritis	0	1
Oligoarthritis, psoriatic arthritis, undifferentiated arthritis	0.34	1.40 (0.91–2.16)

* The 2‐year, 4‐year, and 7‐year baseline survival probabilities are 0.94, 0.91, and 0.90, respectively; the mean linear predictor is −0.71. See Table 1 for definitions.

† Indicates statistically significant.

### External validation and recalibration of prediction model

**Figure 1:**
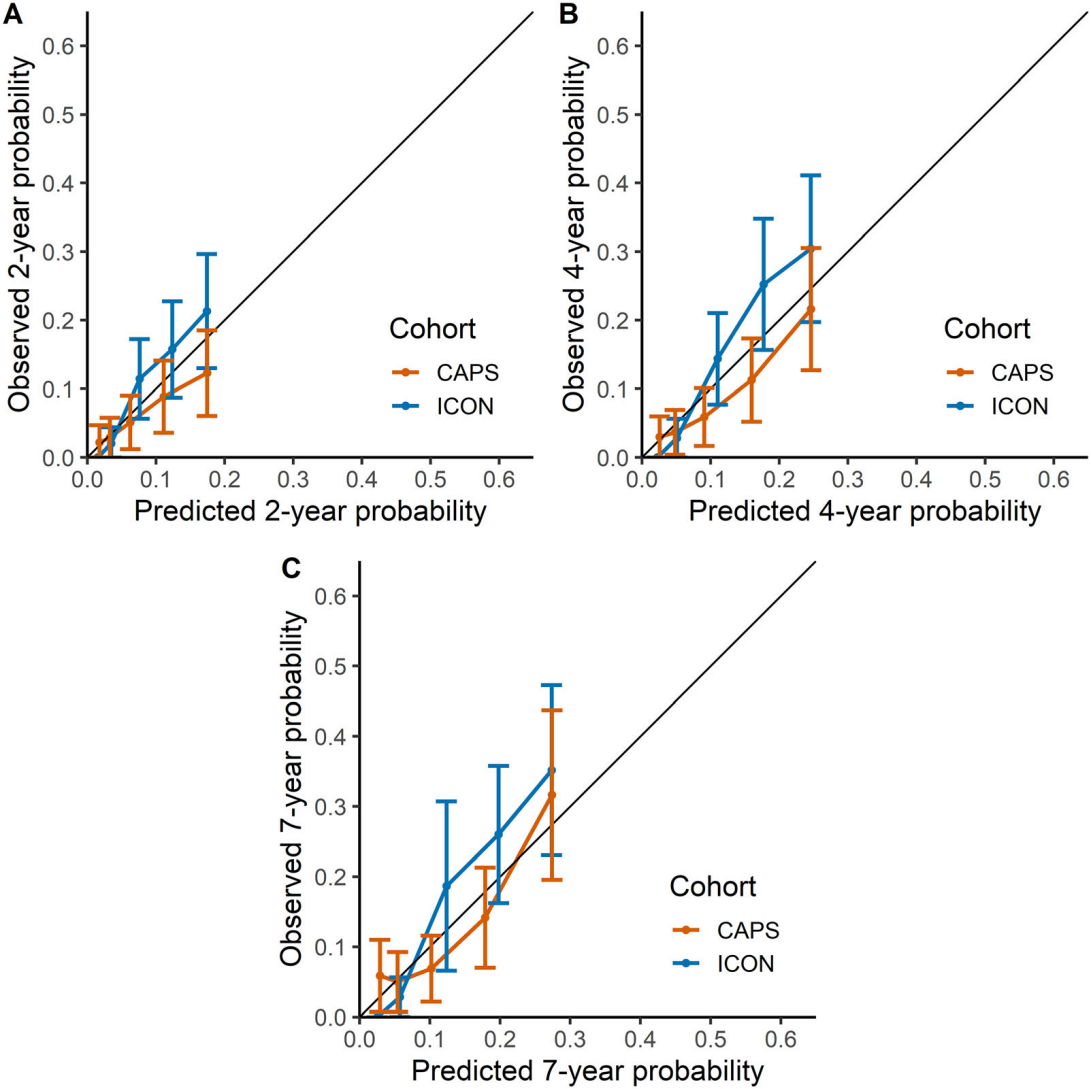
Plots of the calibrated prediction model for predicting new‐onset chronic uveitis in patients with juvenile idiopathic arthritis. Plots represent the observed 2‐year **(A)**, 4‐year **(B)**, and 7‐year **(C)** probabilities/Kaplan‐Meier estimates of new‐onset chronic uveitis versus the mean predicted probabilities within quintiles of the Childhood Arthritis Prospective Study (CAPS) and Inception Cohort of Newly diagnosed patients with juvenile idiopathic arthritis (ICON) validation cohorts. Whiskers represent the 95% confidence intervals.

The C statistics of the prediction model for the 2‐, 4‐, and 7‐year risk of uveitis in the CAPS and ICON cohorts ranged from 0.67 (95% CI 0.59–0.74) to 0.75 (95% CI 0.72–0.79). These were slightly higher than the C statistics of a model with parameters used in the Heiligenhaus screening recommendations (Table 4). Based on calibration plots, the overall best performing model was obtained by incorporating the 2‐, 4‐, and 7‐year baseline survival probabilities from the ICON cohort into the prediction model (Figure 1). The resulting 2‐, 4‐, and 7‐year baseline survival probabilities were 0.94, 0.91, and 0.90, respectively. The formula of this calibrated model for calculating a predicted probability of developing uveitis in an individual JIA patient is as follows:

$$P\left(\text{chronic uveitis}\right)=1-S_{0}{\left(t\right)}^{\exp\left(0.46\times \mathrm{ANA}\;\text{status}-0.19\times \mathrm{age}\;\mathrm{at}\;\mathrm{JIA}\;\text{onset}+0.34\times \text{ILAR category}+0.71\right)}$$

Variables used in this formula are the baseline survival probability (S_0_), ANA status (1 = positive, 0 = negative), age at JIA onset in years, and ILAR category (1 = oligoarthritis, psoriatic arthritis, or undifferentiated arthritis, 0 = RF– polyarthritis). Different baseline survival probabilities are used for different predictions, i.e., for obtaining the 2‐year risk of uveitis, the 2‐year baseline survival probability should be inserted in the formula.

**Table 4:** Performance of new‐onset chronic uveitis prediction model and Heiligenhaus screening recommendations in validation cohorts*

	Prediction model		Heiligenhaus screening recommendations	
	CAPS cohort	ICON cohort	CAPS cohort	ICON cohort
2‐year uveitis risk	0.67 (0.59–0.74)	0.74 (0.69–0.78)	0.65 (0.58–0.75)	0.70 (0.65–0.74)
4‐year uveitis risk	0.69 (0.63–0.76)	0.75 (0.71–0.78)	0.69 (0.63–0.75)	0.71 (0.67–0.74)
7‐year uveitis risk	0.70 (0.64–0.76)	0.75 (0.72–0.79)	0.70 (0.64–0.75)	0.71 (0.68–0.75)

* Values are C statistic (95% confidence interval). CAPS = Childhood Arthritis Prospective Study; ICON = Inception Cohort of Newly diagnosed patients with juvenile idiopathic arthritis.

**Figure 2:**
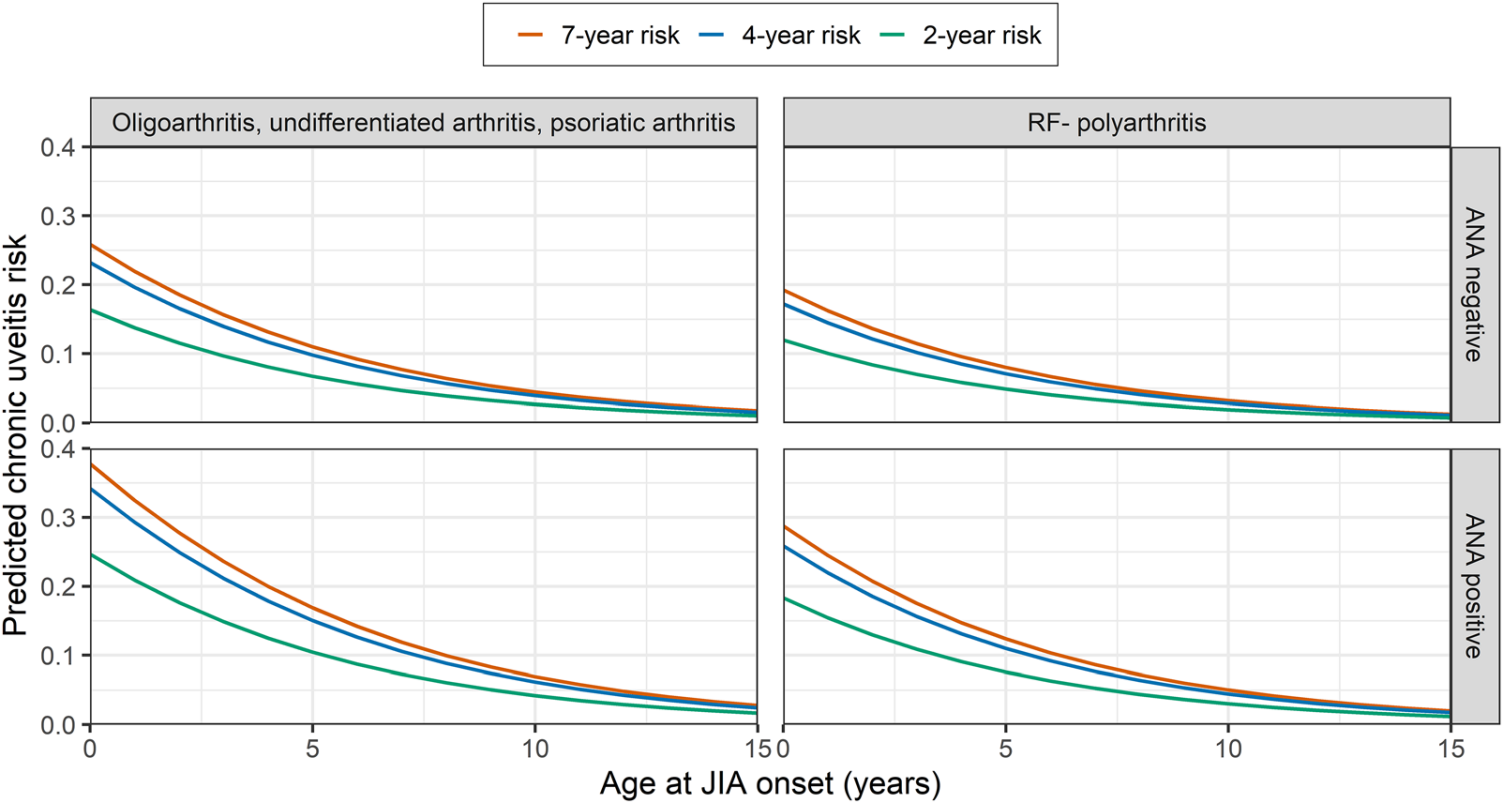
Diagram of cumulative predicted probabilities from the calibrated prediction model for predicting new‐onset chronic uveitis in patients with juvenile idiopathic arthritis (JIA). To read this diagram, identify the patient's International League of Associations for Rheumatology JIA category (across the top) and antinuclear antibody (ANA) status (down the right) to select the appropriate plot. The predicted probability of chronic uveitis (vertical axis) is plotted as a function of the patient's age at JIA onset (horizontal axis). The 2‐, 4‐, or 7‐year risk denotes the risk of developing chronic uveitis in the 2, 4, or 7 years after onset of JIA. Color figure can be viewed in the online issue, which is available at http://onlinelibrary.wiley.com/doi/10.1002/art.42329/abstract.

For clinical practice, a diagram is provided from which the cumulative 2‐, 4‐, and 7‐year risk of new‐onset chronic uveitis can be determined as a function of the predictor variables (Figure 2). Predictions can also be obtained from a digital risk calculator (Supplementary Table 3, available on the *Arthritis & Rheumatology* website at https://onlinelibrary.wiley.com/doi/art.42329).

## DISCUSSION

In this study, we developed and externally validated a prediction model for new‐onset chronic uveitis in JIA patients. Using this model, individual risk estimates for chronic uveitis can easily be obtained from a diagram or risk calculator. Predictions following this model could be used by pediatric rheumatologists to more accurately inform patients and parents and might provide rationale for therapy with ADA or infliximab instead of etanercept for JIA. In addition, these predictions have the potential to guide clinicians in determining screening frequency.

The variables in the prediction model are common clinical parameters in the management of JIA, making our model applicable for clinical practice worldwide. Several studies have shown that ANA status and age at JIA onset are associated with the risk of developing uveitis in JIA (5, 26, 27, 28), and current ophthalmologic screening guidelines also incorporate these factors (4, 7, 9, 10, 11). Previous studies have suggested sex differences in risk factors for uveitis in JIA (29), but in the current study, the same model predictors were selected when restricting analyses to only boys or only girls. The decision to group together psoriatic arthritis, undifferentiated arthritis, and oligoarthritis was based on 2 large studies which found that RF– polyarthritis patients have a lower risk of developing uveitis compared to this group of patients (9, 17).

Since we want our model to be able to provide risk estimates for uveitis early in the disease course of JIA, we decided not to distinguish between persistent and extended oligoarthritis, given that the latter diagnosis might take years to become obvious. For the same reason, we did not consider drug therapy for inclusion in the prediction model. Nevertheless, for the Pharmachild and ICON cohorts, we observed that JIA patients who did not develop uveitis more often took MTX and ADA than patients who did develop uveitis, suggesting a protective effect. This effect might be further supported by the short duration from last MTX stop to uveitis onset that was observed in the Pharmachild cohort (median 0.9 years). Several other studies have reported evidence for a protective effect of MTX and ADA against the development of uveitis in JIA (26, 30, 31, 32, 33).

Geographic residence was significantly associated with uveitis in the Pharmachild cohort, with Western European residence being a significant risk factor, which is consistent with the literature (15). However, addition of this variable to the prediction model resulted in C statistics of 0.37 and 0.39 in the ICON and CAPS cohorts, respectively. This can probably be attributed to unstable coefficient estimates due to the high heterogeneity in the “other region” group, and the fact that there were no patients from Germany or the UK in the Pharmachild cohort.

This study provides the first validated tool for predicting chronic uveitis at different JIA disease durations in an individual JIA patient. One previous study provided a prediction model for uveitis in JIA patients, but this model did not discriminate between acute and chronic uveitis, did not incorporate disease duration, and was not externally validated (17). Another study reported a model for chronic uveitis, but this model also did not incorporate disease duration, lacked external validation, and only included RF– polyarthritis and oligoarthritis patients (34). Calibration and discrimination of our model in the validation cohorts was satisfactory. This demonstrates that the model is capable of predicting the risk of uveitis in JIA patients from settings other than Pharmachild. For instance, patients from the CAPS and ICON validation cohorts were more often ANA positive than patients from the Pharmachild cohort. This could be partly caused by different methodologies for ANA testing in different countries but is most probably the result of a difference in oligoarthritis prevalence, which is known to be higher in Western European countries compared to the rest of the world (1).

The calibration plots revealed that the majority of model predictions for uveitis in the CAPS cohort were slight overestimations, whereas predictions in the ICON cohort were slight underestimations. This is probably caused by the larger uveitis prevalence in ICON (15.7%) compared to CAPS (12.6%). Nonetheless, the prevalence of uveitis in both validation cohorts corresponds to the range of prevalence rates reported in the literature (3). Furthermore, our model had higher discriminative power in the ICON cohort compared to the CAPS cohort, likely due to differences in ILAR categories of patients who developed uveitis; 17% of patients with psoriatic arthritis, undifferentiated arthritis, or oligoarthritis in the ICON cohort developed uveitis versus 13% of these patients in the CAPS cohort.

The cumulative 2‐, 4‐, and 7‐year predicted risks for uveitis following our recalibrated model reveal that the immediate risk of developing chronic uveitis decreases with increasing JIA disease duration. For example, the 7‐year predicted risk is only slightly larger than the 4‐year predicted risk. This is consistent with earlier evidence on the relationship between JIA disease duration and risk of uveitis (9, 35, 36, 37, 38). Nevertheless, since the number of censored patients for deriving a 7‐year risk is higher than the number of censored patients for deriving a 2‐year risk, it is not recommended to use our model to obtain a “remaining risk” for uveitis as a function of the JIA disease duration of a patient. Instead, our model is most valid when applied at first presentation with JIA. Also, as evidenced by the C statistics and calibration plots, our model performs better in predicting long‐term risks than short‐term risks.

The prediction model had higher discriminative power in both validation cohorts than a model based on parameters from the commonly used Heiligenhaus screening recommendations (9). This screening guideline uses a cutoff value of 6 years for age at JIA onset and does not distinguish between psoriatic, oligoarticular, undifferentiated, and RF– polyarticular JIA. Nevertheless, the performance of the Heiligenhaus parameters in both validation cohorts was acceptable, with C statistics of 0.70 and 0.71 for the 7‐year predicted uveitis risk. Therefore, these guidelines remain suitable for ophthalmologic screening of JIA patients and need not be replaced by our prediction model. However, 1 advantage of our prediction model over these screening guidelines is the ability to obtain/provide absolute risk estimates instead of subjective “high”, “low”, or “moderate” risk categories.

Based on the prediction model, we propose a set of points for improving the current standard of care in JIA patients with regard to uveitis development. Given the high predicted uveitis risk for ANA‐positive patients with oligoarthritis, psoriatic arthritis, or undifferentiated arthritis and age at JIA onset ≤6 years, screening for uveitis once every 2 months during the first year after JIA onset, once every 3 months during the second year, and once every 4 months during the third and fourth years seem appropriate. Indeed, it has been suggested to increase uveitis screening frequency to once every 2 months in the highest risk group of JIA patients (39). Also, screening for uveitis once every 4 months during the first 2 years after JIA onset and once every 6 months during the next 2 years could be considered for ANA‐positive patients with oligoarthritis, psoriatic arthritis, or undifferentiated arthritis and age at JIA onset >6 years. ANA‐negative patients with oligoarthritis, psoriatic arthritis, or undifferentiated arthritis and age at JIA onset ≤6 years might be screened once every 4 months during the first 4 years. Based on our model, it could also be useful to differentiate between RF– polyarthritis and oligoarthritis, psoriatic arthritis, or undifferentiated arthritis when determining screening frequency, which is not reflected in the Heiligenhaus screening recommendations. These suggestions will be discussed in the Multinational Interdisciplinary Working Group for Uveitis in Childhood, with the aim of tailoring uveitis screening in JIA using evidence‐based medicine. Apart from modifying screening frequency, low predictions for uveitis according to our model could also be used to comfort patients and parents. For example, a pediatric rheumatologist could inform parents about the relatively low risk their child will develop uveitis. Finally, our model suggests an advantage to starting MTX or even ADA therapy instead of intraarticular injections in JIA patients with high predicted risks for uveitis, which we define as ≥15%.

This study has limitations. First, for a large number of uveitis patients in the Pharmachild registry, no diagnosis date was available. Therefore, these patients had to be excluded, and the resulting prediction model had to be recalibrated using the ICON validation cohort. We observed that uveitis patients without a diagnosis date more often had oligoarthritis and were ANA positive. Yet, the recalibrated prediction model performed well in the validation cohorts. Also, multivariable logistic regression analysis in the Pharmachild cohort including uveitis patients without a diagnosis date yielded the same predictor variables. Furthermore, the majority of included patients were treated in tertiary care centers. Therefore, it is uncertain how our model would perform in JIA patients who are seen in centers without ample experience caring for JIA and uveitis patients who have low disease activity and do not receive DMARDs, for which additional recalibration might be needed.

A great strength of the present study is the large sample size of the Pharmachild model development data, with patients from multiple countries and the use of inception cohorts from further geographic settings for validation. The latter is ideal for studying early‐onset uveitis in JIA.

Future practical recommendations for health care providers and patients based on our model should be jointly formulated by clinicians and patients and endorsed by organizations such as the European Reference Network on immunodeficiency, autoinflammatory and autoimmune diseases. In addition, further research is needed to evaluate how the use of our model in clinical practice affects management and outcomes of JIA patients. Unfortunately, such research on clinical application of models is rarely performed (40). In addition, the current model could be extended with relevant biomarker data. Studies have highlighted an elevated erythrocyte sedimentation rate, calcium‐binding protein S100A12, and HLA–DRB1*11 in girls as potential predictive factors (26, 29, 38, 41).

To conclude, we provide for the first time a validated prediction model for new‐onset chronic uveitis at different JIA disease durations in an individual JIA patient. Predictions using this model can easily be obtained from common clinical parameters.

## AUTHOR CONTRIBUTIONS

All authors were involved in drafting the article or revising it critically for important intellectual content, and all authors approved the final version to be published. Joeri van Straalen had full access to all of the data in the study and takes responsibility for the integrity of the data and the accuracy of the data analysis.

### Study conception and design

van Straalen, de Roock, Swart.

### Acquisition of data

Minden, Heiligenhaus, Hyrich, Lamot, Olivieri, Gallizzi, Smolewska, Faugier, Pastore, Hashkes, Herrera, Emminger, Consolini, Wulffraat, Ruperto, Swart.

### Analysis and interpretation of data

van Straalen, Kearsley‐Fleet, Klotsche, de Boer, Wulffraat, Ruperto, Swart.

## Supporting information

Disclosure Form

**Supplementary Figure S1** Selection of participants in Pharmachild.

**Supplementary Table 1** Overview of countries of included patients and corresponding geographical regions.

**Supplementary Table 2** Characteristics of chronic uveitis cases with and without available diagnosis date in the Pharmachild cohort.

**Supplementary Table 3** Risk calculator for chronic uveitis in juvenile idiopathic arthritis.

## Data Availability

Pharmachild, CAPS, ICON, and all participating centers obtained approval from their respective ethics committees, and each study was conducted in accordance with the Declaration of Helsinki. All patients provided written informed consent/assent based on existing national regulations. All relevant data are reported in the article. Additional details can be provided by the corresponding author upon reasonable request.
