# Supplementary figures and images for: Development and External Validation of a Model Predicting New‐Onset Chronic Uveitis at Different Disease Durations in Juvenile Idiopathic Arthritis
Source: Arthritis Rheumatol. 2022 Dec 13;75(2):318–27. doi: 10.1002/art.42329 (PMC10108055; doi:10.1002/art.42329)

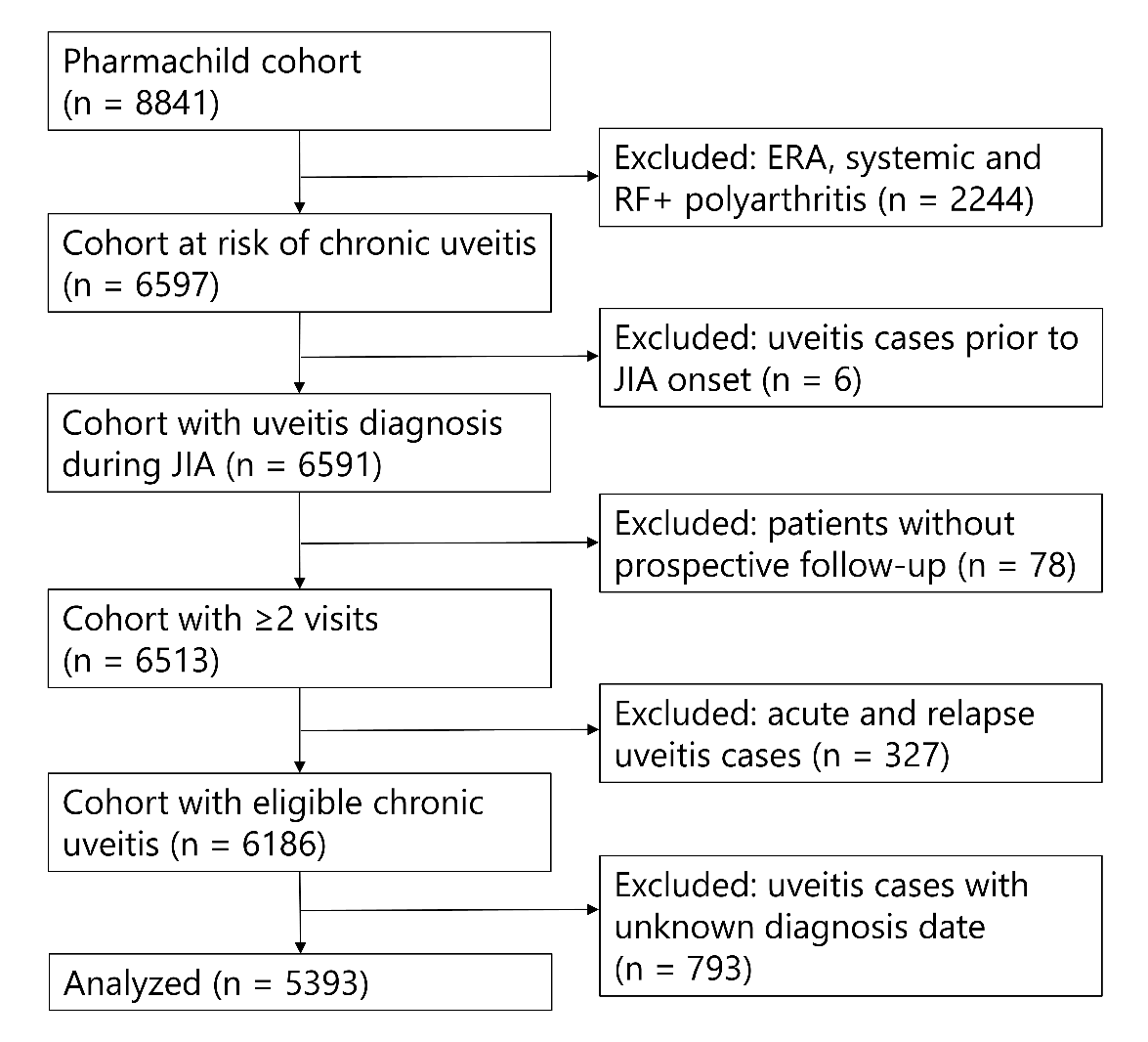


**Supplementary Figure S1. Selection of participants in Pharmachild.**

Supplement: Supplementary file 2 — Supplementary Figure S1 Selection of participants in Pharmachild. [file ART-75-318-s002.docx]
