# Supplementary material for: Development and External Validation of a Model Predicting New‐Onset Chronic Uveitis at Different Disease Durations in Juvenile Idiopathic Arthritis
Source: Arthritis Rheumatol. 2022 Dec 13;75(2):318–27. doi: 10.1002/art.42329 (PMC10108055; doi:10.1002/art.42329)
Supplement: Supplementary file 3 — Supplementary Table 1 Overview of countries of included patients and corresponding geographical regions. [file ART-75-318-s003.docx]

Supplementary Table 1. Overview of countries of included patients and corresponding geographical regions.

| **Geographical region** | **Countries** |
| --- | --- |
| Southern Europe | Greece, Italy, Spain |
| Central and Eastern Europe | Bulgaria, Croatia, Czech Republic, Hungary, Latvia, Lithuania, Poland, Romania, Serbia, Slovakia, Russia, Slovenia |
| Western Europe | Austria, France, Netherlands, Switzerland |
| Scandinavia | Denmark, Norway |
| Other | Israel, Libya, Oman, Saudi Arabia, Turkey, Argentina, Brazil, Peru, Ecuador, Mexico, India, Singapore |
