# Supplementary material for: Development and External Validation of a Model Predicting New‐Onset Chronic Uveitis at Different Disease Durations in Juvenile Idiopathic Arthritis
Source: Arthritis Rheumatol. 2022 Dec 13;75(2):318–27. doi: 10.1002/art.42329 (PMC10108055; doi:10.1002/art.42329)
Supplement: Supplementary file 4 — Supplementary Table 2 Characteristics of chronic uveitis cases with and without available diagnosis date in the Pharmachild cohort. [file ART-75-318-s001.docx]

Supplementary Table 2. Characteristics of chronic uveitis cases with and without available diagnosis date in the Pharmachild cohort.

| **Characteristics** | **Chronic uveitis with diagnosis date  (n = 107)** | **Chronic uveitis  without diagnosis date (n = 793)** |
| --- | --- | --- |
| Girls, n (%) | 82 (76.6%) | 632 (79.7%) |
| Age at JIA onset (years),  median (IQR) | 2.2 (1.6 – 4.1) | 2.6 (1.7 – 4.4) |
| ILAR category, n (%) |  |  |
| Oligoarthritis | 58 (54.2%) | 531 (67.0%) |
| Polyarthritis RF - | 29 (27.1%) | 173 (21.8%) |
| Psoriatic arthritis | 8 (7.5%) | 27 (3.4%) |
| Undifferentiated arthritis | 12 (11.2%) | 62 (7.8%) |
| Laboratory characteristics, n (%) |  |  |
| ANA positive | 68 (63.6%) n = 107 | 560 (72.2%) n = 776 |
| RF positive | 0 (0.0%) n = 91 | 2 (2.9%) n = 692 |
| HLA-B27 positive | 9 (14.8%) n = 61 | 52 (12.1%) n = 430 12.1%) |
| IQR = interquartile range, n = number | | |
